# A novel variant of genotype 7b hepatitis C virus emphasizing viral hepatitis elimination challenges for sub-Saharan Africa

**DOI:** 10.11604/pamj.2020.36.232.24726

**Published:** 2020-07-30

**Authors:** Mark Wayne Sonderup, Heidi Smuts, Catherine Wendy Spearman

**Affiliations:** 1 Division of Hepatology, Department of Medicine, Faculty of Health Sciences, University of Cape Town and Groote Schuur Hospital, Cape Town, South Africa,; 2 Division of Medical Virology, National Health Laboratory Service, Groote Schuur Hospital, Cape Town, South Africa

**Keywords:** Hepatitis C, genotype diversity, elimination programmes

## Abstract

Sub-Saharan Africa has approximately 10.15 million people viraemic with chronic hepatitis C virus infection, extensive genotype and sub-genotype diversity is present, in addition to novel hepatitis C genotypes. Many of the unusual genotypes have extensive baseline resistance associated substitutions with direct acting antiviral therapy treatment outcome data, limited. We report a patient found to have a novel genotype 7b variant with extensive baseline resistance associated substitutions. There is a clear need for a better understanding of the virological characteristics of hepatitis C populations in sub-Saharan Africa to guide best optimal treatment decisions in national hepatitis C elimination programmes.

## Introduction

Sub-Saharan Africa has approximately 14% (˜10.15 million) of the global 71 million people viraemic with chronic hepatitis C virus (HCV) infection [1]. A key feature of HCV in the region is genotype and extensive sub-genotype diversity, with genotype 1 through 5 documented, but regional clustering of certain genotypes occurring e.g. genotype 1 and 4 [2]. Recent data from Uganda and the Democratic Republic of Congo (DRC) has highlighted this sub-genotype diversity as well as finding previously described novel HCV genotypes [3]. An important observation was the wide range of resistance associated substitutions (RAS) observed, especially in novel genotypes. Given limited access to direct acting antiviral (DAA) HCV therapy, treatment outcome data in these patients was not known. In terms of novel HCV genotypes, genotype 7 HCV was first reported in 2015 in four patients from the DRC [4]. It was genetically distinct from genotypes 1 to 6, fulfilled criteria as a new genotype and the prototype sequence (accession number NC_030791/EF108307) was classified as subtype 7a.

In 2016, using next generation sequencing, a genotype 7b subtype was identified in a man from Kinshasa, in the DRC [5]. Similarly a wide range of RAS were detected in both the NS3, NS5A and NS5B of the virus. As treatment outcome data did not exist, the influence of these RAS are unknown. Treatment data for novel HCV genotypes is scarce. In the ASTRAL-1 study evaluating 12 weeks of the combination pan-genotypic DAA therapy sofosbuvir and velpatasvir, a patient originally from the DRC and enrolled as genotype 2a, achieved a sustained virological response (SVR) [6]. During a post-trial analysis, the patient was confirmed as having genotype 7a with phylogenetic analysis demonstrating it to most closely resemble the previously identified genotype 7a patients [7]. Baseline RAS data for this patient is unpublished and not accessible. What is known is that the subtype diversity seen in genotypes 1 and 4 in sub-Saharan Africa does influence SVR rates. This has been observed in recent treatment data that has highlighted reduced responsiveness to standard DAA regimens, notably in genotype 1 (non-1a or 1b subtypes) and in genotype 4, notably subtype 4r [8, 9]. Many of the treatment regimens included the generically available and cost effective NS5A-NS5B DAA combinations of daclatasvir and sofosbuvir or ledipasvir and sofosbuvir.

## Patient and observation

**Figure 1:**
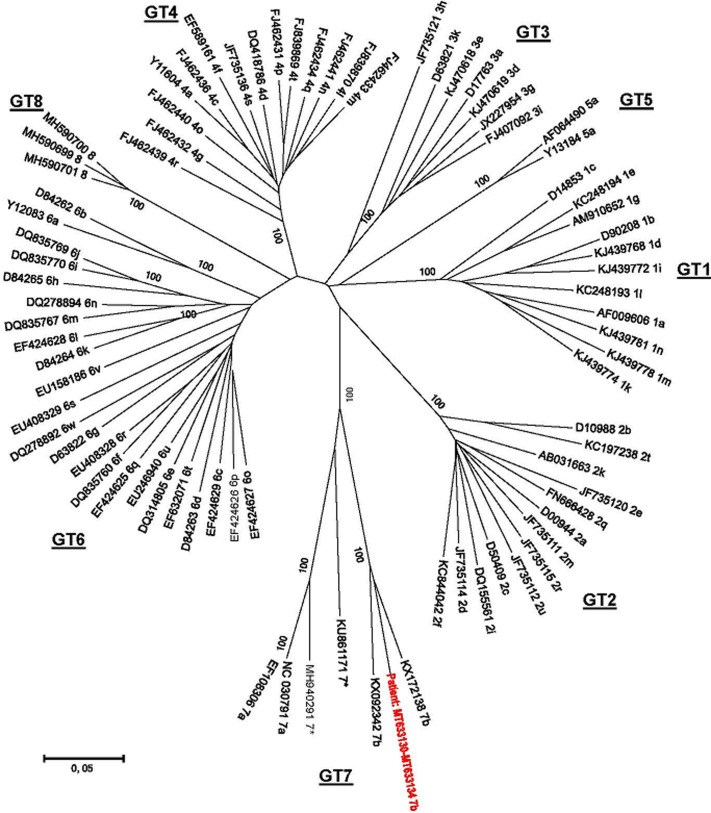
phylogenetic NS5B radial tree comparison of patient (in red) assigned as a novel genotype 7b variant

In 2019, a 66-year-old woman from Luanda, Angola was referred to our service. Apart from well controlled hypertension, she was well with no other co-morbidities. Chronic HCV infection was diagnosed 10 years prior, following routine health screening. At that point, genotype analysis performed in Angola reported genotype 2a infection. At that point, she had declined pegylated interferon and ribavirin treatment given its potential toxicities. The route of acquisition of her HCV infection was unknown as no clear identifiable risk factors were evident. Her ALT was 71 IU/L (laboratory normal range 5-40 IU/L) and significant liver fibrosis was excluded with a FibroScan^R^liver stiffness measurement of 7.6kPa and an AST to Platelet Ratio Index (APRI) score of 0.2. Liver ultrasound was normal and HCV viral load was 9 772 784 IU/mL (Xpert, Cepheid, CA, USA). HIV and HBsAg testing, was negative. We did baseline population-based Sanger sequencing to evaluate for RAS in the NS3, NS5A and NS5B HCV genes using pan genotypic primers and the web-based geno2pheno program (Table 1). Sequences were aligned with GenBank reference sequences using BioEdit and a NS5B neighbour-joining phylogenetic tree was constructed in Mega 6.0 with 1000 bootstrap resamplings (Figure 1). We assigned the sequence as a novel genotype 7b variant (accession number MT633130-MT633134). Considering the presence of extensive baseline RAS in the patient, a DAA therapy regimen was considered (Table 1).

**Table 1:** baseline NS3, NS5A and NS5B resistance associated substitutions (RAS)

Gene	Location	GT7b RAS
NS3	T54	S
NS3	Q80	E
NS3	A156	K
NS3	D168	Q
NS5A	M28	L
NS5A	Q30	F
NS5A	Y93	H
NS5B	M414	A
NS5B	S453	T
NS5B	V553	F
NS5B	R554	S
NS5B	E557	G
NS5B	D561	I
NS5B	P565	F

The only available data on treatment was the single genotype 7a patient inadvertently treated in the ASTRAL-1 study [6]. A sofosbuvir/velpatasvir combination was considered, however given the range of RAS, particularly the NS5A 93H mutation, the choice of velpatasvir posed a concern. This mutation impairs treatment response since it induces a high fold in vitro resistance to daclatasvir and velpatasvir. This has been observed to negatively influence SVR in real world experience with DAA therapy, mostly with genotype 3 HCV [10]. An obvious lack of data of the NS5A 93H RAS in genotype 7b in addition to the range of other RAS present, influenced our decision making. Similarly and although not analogous to our patient, data observed in the retreatment MAGELLAN-1 part 2 study suggested that a 16 week duration of treatment with the NS3/NS5A DAA combination of glecaprevir and pibrentasvir, to be better than 12 weeks in patients with prior DAA failure and significant RAS [11]. Given this, we elected to treat the patient with 16 weeks of glecaprevir and pibrentasvir combination as well as the NS5B nucleotide polymerase inhibitor sofosbuvir, all as single daily dosing. After 4 weeks on therapy, HCV viral load was recorded at <10IU/ml (HCV Xpert, Cepheid, CA, USA) and undetected at the end of treatment. Sustained virological response at 12 weeks post end of treatment (SVR12), was confirmed.

## Discussion

We report for the first time an HCV genotype 7b variant. Although this variant is newly described, the patient also underscores challenges in sub-Saharan Africa where a limited, but growing body of experiential treatment data, suggests that prevailing genotype and subtype diversity in countries, may negatively impact SVR rates. This has invariably been observed with standard DAA HCV treatment regimens that notably, are generically available, cost effective and important in building national HCV elimination programmes. Conceding that the DAA regimen used in our patient was possibly overtly aggressive, the patient does represent the dual challenge. Firstly, that there are sub-populations of HCV in sub-Saharan Africa that are less likely to achieve SVR with standard treatment regimens. We have very little knowledge of these unique genotypes and how they are likely to respond to standard therapies. The extensive baseline RAS present in this patient suggests they may be less likely to achieve SVR. Secondly, in our resource-constrained setting, opportunities for re-treatment in the event of non-SVR are problematic and limited by availability, access and cost. Achieving treatment success, the first time around, is crucial. We therefore support the need and would suggest that sub-Saharan African countries sample their HCV population to determine more accurate genotype and sub-genotype characteristics. Such data may serve for better planning in guiding best treatment practice for expanded elimination programmes and thereby potentially limit patients not achieving SVR.

## Conclusion

We report a patient with a unique HCV genotype 7b variant. The case provides insight into the extensive HCV genotype diversity in sub-Saharan Africa and highlights the suggested need for countries to gain a better understanding of their HCV populations while developing and implementing national HCV treatment programmes.
